# Fulminant hepatitis due to very severe sinusoidal obstruction syndrome (SOS/VOD) after autologous peripheral stem cell transplantation: a case report

**DOI:** 10.1186/s13104-018-3533-0

**Published:** 2018-07-03

**Authors:** Emmanuelle Tavernier, Emilie Chalayer, Jérôme Cornillon, Anne Pouvaret, Jean-Alain Martignoles, François Casteillo, Jérémy Terreaux, Elisabeth Daguenet, Denis Guyotat

**Affiliations:** 1 Department of Clinical Hematology, Institut de Cancérologie Lucien Neuwirth, 108 Bis Avenue Albert Raimond, 42270 Saint Priest en Jarez, France; 2 Department of Pathology, University Hospital, Saint Etienne, France; 3 Intensive Care Unit, University Hospital, Saint Etienne, France

**Keywords:** Mantle cell lymphoma, Autologous transplantation, Sinusoidal obstruction syndrome, BEAM regimen, Oxaliplatin, Case report

## Abstract

**Background:**

Hepatic veno-occlusive disease, also called sinusoidal obstruction syndrome (SOS/VOD), is a potentially fatal complication of allogeneic or autologous hematopoietic stem cell transplantation. A plethora of transplant and patient-related risk factors predispose to SOS/VOD and should be taken into account for prognosis assessment as well as for adequate therapeutic intervention.

**Case presentation:**

We describe the case of a mantle cell lymphoma patient who developed a fulminant hepatitis following oxaliplatin-containing intensive chemotherapy and autologous transplantation. This clinical manifestation was secondary to a very severe SOS/VOD. The patient did not exhibit the usual risk factors and presented a non-classical form with major cytolysis, thus puzzling SOS/VOD diagnosis in this context.

**Conclusion:**

SOS has been previously reported after oxaliplatin-based chemotherapy regimens for colorectal cancers, in particular in patients with colorectal liver metastases. We therefore suspected a potential relationship with oxaliplatin-based regimen as a driver of SOS/VOD in a non-susceptible lymphoma patient. With regards to this case, clinicians and especially intensivists should be aware of this atypical presentation.

## Background

Sinusoidal obstruction syndrome or veno-occlusive disease (SOS/VOD) is a life-threatening complication [1] following hematopoietic stem cell transplantation (HSCT) with median incidence lower than 10% after autologous HSCT [2]. SOS/VOD pathophysiology results from sinusoidal endothelial cells damage and hepatic injury due to toxic metabolites generated by conditioning chemotherapy and radiotherapy [3]. We presently report the case of a patient who developed a very severe SOS/VOD, as revealed by an unusual increase of transaminase level.

## Case presentation

A 59-year-old and overweight man (weight = 87 kg, BMI = 29.75) who suffered from a sleep apnea syndrome, was diagnosed with a stage III mantle cell lymphoma in December 2014. On admission, he presented systemic lymphadenopathy without any bone marrow involvement. Laboratory tests showed normal liver enzymes levels as well as negative hepatitis B and C serological profiles.

**Fig. 1:**
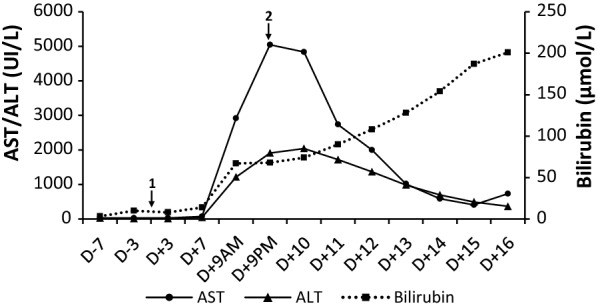
Laboratory data during the clinical course from Day −7 (D −7) to Day +16 (D +16): hepatic enzymes with AST, ALT and total bilirubin. The reference day corresponds to the autologous transplantation (Day 0, depicted with the arrow 1). Defibrotide treatment was initiated on Day +9 in the evening (D +9PM, depicted with the arrow 2)

**Fig. 2:**
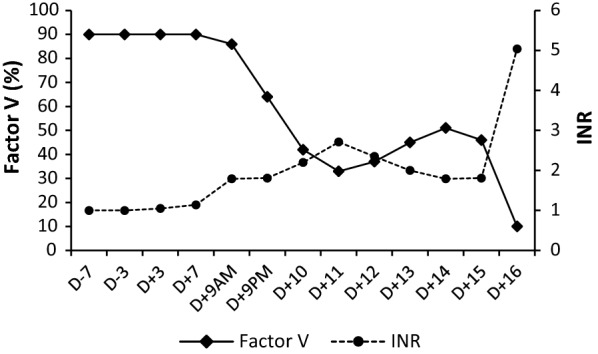
Laboratory data during the clinical course from Day −7 (D −7) to Day +16 (D +16): coagulation factor with Factor V and INR

An oxaliplatin-based polychemotherapy followed by high-dose therapy and autologous stem cell transplantation was proposed. First-line chemotherapy with four cycles of R-DHAX regimen, including rituximab, dexamethasone, cytarabine and oxaliplatin was administered. After three courses, PET-CT (positron emission tomography) response assessment indicated a complete metabolic response. The patient then received conditioning regimen with BEAM 400, consisting of bicnu (300 mg/m^2^) for 1 day, etoposide (400 mg/m^2^) combined to cytarabine (400 mg/m^2^) for 4 days and melphalan (140 mg/m^2^) for 1 day prior to autologous HSCT. Anti-infective prophylaxis included valacyclovir and fluconazole, starting on Day −7. On March 16th, 2015 (Day 0), 9.8 × 10^6^ cells CD34+/kg were infused. During this procedure, laboratory data did not display any abnormality, especially hepatic enzymes levels that were within the normal range. Despite antimicrobial therapy with Piperacillin–Tazobactam, the patient had persistent fever over the ensuing 72 h, requiring an empiric antifungal treatment with Voriconazole. No signs of invasive aspergillosis were detected (normal CT-scan) and aspergillus antigenemia were negative. Voriconazole was then replaced by Caspofungin on Day +6. On Day +8, oligoanuria was observed and physical examination revealed hepatomegaly, fluid retention, ascites and weight gain < 5% (i.e. 90 kg, +2 kg/48 h). Additionally, thrombocytopenia refractory to platelet transfusion was noted. On Day +9, the serum transaminase concentration increased in an explosive manner from 75 to 2914 UI/L in the morning and 5046 UI/L in the evening for aspartate aminotransferase (AST) and from 45 to 1216 UI/L for alanine aminotransferase (ALT). Similarly, the bilirubin level had reached 67 µmol/L (N < 21) accompanied with elevated creatinine level shifting from normal value to 230 µmol/L within 24 h (Fig. 1). Alkaline phosphatase levels were initially normal and progressively increased along with SOS/VOD syndrome (2× ULN). The patient developed coagulation disorders as revealed by a progressive increase of INR whereas the Factor V remained normal (Fig. 2). An elevated level of plasminogen activator inhibitor (PAI-1) was also noted with a value of 51 UA/mL (N < 16). An abdominal ultrasound was performed and confirmed hepatomegaly, ascites and decreased velocity in the portal venous flow. The subject whose weight increased more than 5% (i.e. 92 kg) was therefore diagnosed with hepatic VOD and was admitted to the Intensive Care unit. A treatment with Defibrotide at a dose of 25 mg/kg/day was immediately initiated.

**Fig. 3:**
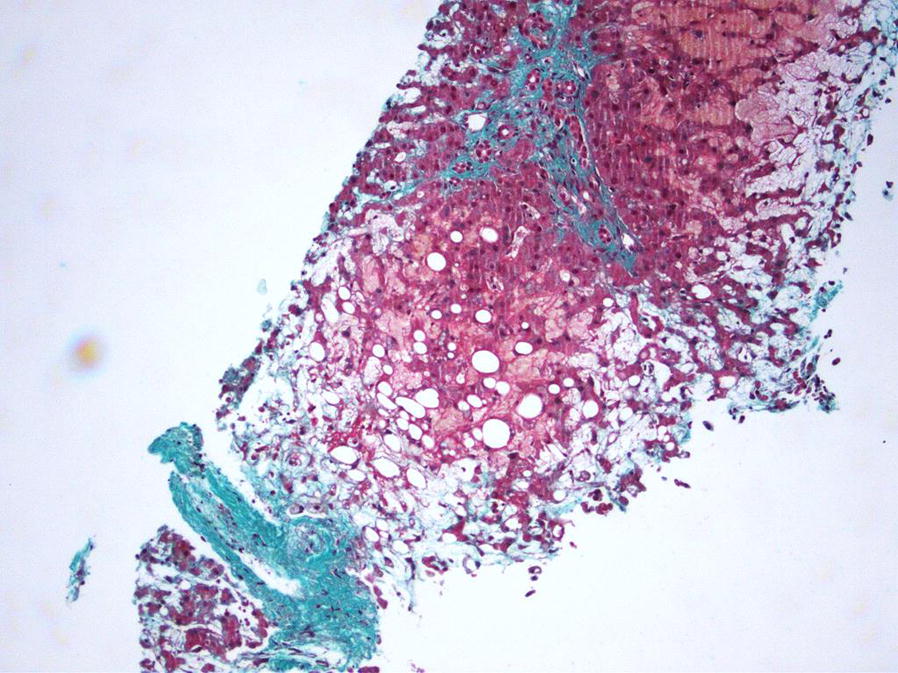
Microscopic finding of the liver biopsy. Veinous dilatation and congestion in veno-occlusive disease with fibrosis and sub-intimal oedema (trichrome stain, ×200)

Because of the atypical major increase in transaminases levels, a transvenous liver biopsy was performed on Day +10 and showed a patchy perivenular sinusoidal dilatation and congestion with hepatocyte plate disruption (Fig. 3). A second abdominal ultrasound further supported the diagnosis of SOS/VOD with a worsening of doppler parameters, then harboring a reversed hepatic venous flow. Moreover, differential diagnoses such as fulminant viral hepatitis (hepatitis A, B, C and E, herpes simplex virus, cytomegalovirus, Epstein–Barr virus) were excluded. Despite symptomatic measures along with the maintenance of an adequate fluid balance and ascites removal with albumin infusion, renal failure could not be controlled. Because of anuria, hemodialysis was thus implemented on Day +9.

Neutrophils recovery was achieved on Day +11 and liver dysfunction gradually improved from Day +13 to Day +15 with decreased transaminases levels ([AST] 408 UI/L; [ALT] 494 UI/L) (Fig. 1). Although his overall condition was progressively recovering, the patient presented a sudden hemodynamic instability with an acute respiratory distress syndrome requiring mechanic ventilation, on Day +16. A fulminant liver failure with uncontrolled lactic acidosis was observed. An abdominal CT scan was performed and showed an acute mesenteric ischemia with hepatic and renal ischemic injury. The patient died a few hours later of a multi-organ-system failure.

## Discussions and conclusions

SOS/VOD is a dramatic complication observed after HSCT and is associated with a high mortality rate in the most severe forms. This case is of particular interest as it is uncommon. So far, it is the first reported fulminant hepatitis induced by SOS/VOD.

A number of pre-transplant factors and transplant-related risk factors pre-dispose to SOS/VOD occurrence and are well-documented in the literature [4]. In fact, patient and diseases-related features include older age, female gender, Karnofsky score below 90% and advanced disease. Moreover, the risk of SOS/VOD is higher after allo-HSCT by comparison with autologous transplantation. In the current case, the patient did not present the usual risk factors and was not suffering from an underlying liver disease before transplant (normal serum transaminase level, no active viral hepatitis). Besides, the patient who achieved a first complete remission was not heavily pre-treated before autologous HSCT. One can consider about the potential hepatotoxicity of azole antifungal agents. It is yet important to note that the patient was not treated with azole agents at the time of SOS/VOD signs. More interestingly, we can discuss about the possible role of oxaliplatin before transplant. Indeed, SOS has been previously reported after oxaliplatin-based chemotherapy regimens for colorectal cancers, in particular in patients with colorectal liver metastases [5]. Nevertheless, there is no data regarding hepatic toxicity following oxaliplatin-based regimen for lymphoma patients [6, 7]. Even if our patient had not experienced modifications of liver function tests after four cycles of R-DHAX, we therefore suspected that oxaliplatin treatment was a driver for SOS/VOD development in a non-susceptible patient, in a similar way as in patients with colorectal liver metastases.

Overall, this case meets the revised European society for Blood and Marrow Transplantation (EBMT) criteria for diagnosis confirmation of SOS/VOD [4]: occurrence within the first 21 days after HSCT, bilirubin ≥ 2 mg/dL and two other criteria with weight gain > 5% and ascites. The definitive diagnosis was brought by the histology analysis of the liver biopsy. Several publications have further reported the relevance of PAI-1 as a critical marker for the diagnosis of SOS/VOD after HSCT [8, 9]. Interestingly, PAI-1 level was significantly elevated, thus suggesting that PAI-1 is a relevant diagnostic criteria in this case. In addition, results from ultrasound/doppler ultrasonography were consistent with the diagnosis while revealing hepatomegaly, ascites, first decrease in velocity and later reversal of the portal venous flow [10]. Unfortunately, we are missing a measure of the hepatic venous gradient pressure.

It is noteworthy to particularly consider this case because of the kinetics of worsening and the severity of the cytolysis that misled the initial diagnosis. Revised EBMT criteria for severity grading are hence very useful in this situation. Due to the renal failure, the transaminase level > 8× normal and the serum bilirubin level (67 µmol/L), this SOS/VOD was classified as a very severe case, according to the revised EBMT criteria [4]. The kinetics of appearance of the symptoms is of major importance since our patient developed a very severe SOS/VOD within 24 h. Despite early diagnosis and immediate treatment with defibrotide [11–15] as well as a transient improvement of the liver test function, the continuous rising in bilirubin level probably indicates a non-resolving SOS/VOD. A rough degradation was observed on Day +16 and the evolution was fatal due to multi-organ-system failure. Moreover, no evidence of documented sepsis was found that could explain for this sudden deterioration.

Our patient experienced a very severe SOS/VOD without classical risk factors. The diagnosis should be considered in a patient who develops a “fulminant hepatitis” after an intensive chemotherapy since early intervention could improve outcome. One should pay particular attention to this atypical presentation in order to dispense optimal care.

## Abbreviations

SOS: sinusoidal obstruction syndrome
VOD: veno-occlusive disease
HSCT: hematopoietic stem cell transplantation
PET-CT: positron emission tomography
ULN: upper limit of normal
AST: aspartate aminotransferase
ALT: alanine aminotransferase
PAI-1: plasminogen activator inhibitor-1
EBMT: European Society for Blood and Marrow Transplantation
